# Epidemiología genómica de los sublinajes δ del virus SARS-CoV-2 de la segunda ola de COVID en Antioquia en el 2021

**DOI:** 10.7705/biomedica.6862

**Published:** 2024-03-31

**Authors:** Cristian Arbey Velarde, Uriel Hurtado, Andrés Cardona, Celeny Ortiz, Idabely Betancur

**Affiliations:** 1 Laboratorio Departamental de Salud Pública de Antioquia, Secretaría Seccional de Salud y Protección Social de Antioquia, Medellín, Colombia Secretaría Seccional de Salud y Protección Social de Antioquia Secretaría Seccional de Salud y Protección Social de Antioquia Medellín Medellín; 2 Corporación para Investigaciones Biológicas, Medellín, Colombia Corporación para Investigaciones Biológicas Corporación para Investigaciones Biológicas Medellín Medellín; 3 Laboratorio Genómico One Health, Universidad Nacional, Medellín, Colombia Universidad Nacional Universidad Nacional Medellín Medellín; 4 Dirección de Salud Colectiva, Secretaria de Salud de Antioquia, Secretaría Seccional de Salud de Antioquia, Medellín, Colombia Secretaría Seccional de Salud de Antioquia Secretaría Seccional de Salud de Antioquia Medellín Medellín

**Keywords:** SARS-CoV-2, secuenciación genómica, secuencia nucleotídica, vigilancia en salud pública, árbol filogenético, mutación, epidemiología, SARS-CoV-2, genomic sequencing, nucleotide sequence, public health surveillance, phylogenetic tree, mutation, epidemiology

## Abstract

**Introducción.:**

Durante el desarrollo de la pandemia por SARS-CoV-2 en Antioquia se presentaron picos epidemiológicos relacionados con las variantes α, ɣ, β, μ, ƛ y δ, donde δ tuvo la mayor incidencia y prevalencia. Este linaje se considera una variante de preocupación dadas las manifestaciones clínicas que desencadena y sus características epidemiológicas. Se han informado 253 sublinajes δ en la base de datos PANGOLIN. La identificación de estos sublinajes mediante análisis genómico ha permitido rastrear su evolución y propagación.

**Objetivo.:**

Caracterizar la diversidad genética de los diferentes sublinajes δ de SARSCoV-2 en Antioquia y determinar su prevalencia.

**Materiales y métodos.:**

Se recopiló información sociodemográfica de 2.675 muestras y de 1.115 genomas del repositorio GISAID entre el 12 de julio de 2021 y el 18 de enero de 2022. Se seleccionaron 501 por su alto porcentaje de cobertura (>90 %) para realizar análisis filogenéticos e inferencia de frecuencias alélicas de mutaciones de interés.

**Resultados.:**

Se caracterizaron 24 sublinajes donde el más prevalente fue AY.25. En este sublinaje se identificaron mutaciones de interés como L452R, P681R y P681H, que comprendían una frecuencia cercana a 0,99.

**Conclusiones.:**

Este estudio permitió identificar que el sublinaje AY.25 tiene una ventaja de transmisión en comparación con los otros sublinajes δ. Esto puede estar relacionado con la presencia de las mutaciones L452R y P681R que en otros estudios se han visto asociadas con una mayor transmisibilidad, evasión del sistema inmunitario y menor eficacia de los medicamentos contra SARS-CoV-2.

## Abstract (en)

**Introduction.:**

During the development of the SARS-CoV-2 pandemic in Antioquia, we experienced epidemiological peaks related to the α, ɣ, β, μ, ƛ, and δ variants. δ had the highest incidence and prevalence. This lineage is of concern due to its clinical manifestations and epidemiological characteristics. A total of 253 δ sublineages have been reported in the PANGOLIN database. The sublineage identification through genomic analysis has made it possible to trace their evolution and propagation.

**Objective.:**

To characterize the genetic diversity of the different SARS-CoV-2 δ sublineages in Antioquia and to describe its prevalence.

**Materials and methods.:**

We collected sociodemographic information from 2,675 samples, and obtained 1,115 genomes from the GISAID database between July 12^th^, 2021, and January 18^th^, 2022. From the analyzed genomes, 515 were selected because of their high coverage values (>90%) to perform phylogenetic analysis and to infer allele frequencies of mutations of interest.

**Results.:**

We characterized 24 sublineages. The most prevalent was AY.25. Mutations of interest as L452R, P681R, and P681H were identified in this sublineage, comprising a frequency close to 0.99.

**Conclusions.:**

This study identified that the AY.25 sublineage has a transmission advantage compared to the other δ sublineages. This attribute may be related to the presence of the L452R and P681R mutations associated in other studies with higher evasion of the immune system and less efficacy of drugs against SARS-CoV-2.

La epidemia de COVID-19 en Colombia estuvo marcada por cuatro olas de contagio que dejaron 6’369.916 de casos confirmados y 142.780 fallecidos ^1^. En Colombia, el primer caso confirmado de COVID-19 se detectó en Bogotá en un viajero procedente de Milán (Italia) el 6 de marzo del 2020 ^2^. Para el 23 de marzo habían confirmados 314 casos y el 18 de junio el número de contagios detectados y confirmados ascendía a 57.046 con 1.864 muertes ^1^^,^^2^. En Antioquia, el primer caso confirmado se informó el 9 de marzo del 2020 en Medellín ^3^. Entre el 2020 y el 2021, en Antioquia, se registraron más de 790.000 casos confirmados con la presencia de variantes de SARS-CoV-2 tales como α, β, ɣ, δ y ο, siendo δ la más prevalente entre todas las variantes de preocupación ^4^.

La variante δ de SARS-CoV-2 (linaje B.1.617.2 en el sistema PANGO) se identificó por primera vez en India en octubre del 2020. La Organización Mundial de la Salud (OMS) la clasificó por primera vez como una variante de interés el 4 de abril del 2021 y luego su clasificación cambio a variante de preocupación el 11 de mayo del 2021, dada su alta transmisibilidad y la gravedad de los desenlaces clínicos que demostraba ^5^^-^^7^. La propagación global, regional y local de la variante δ se aceleró desde su aparición y desplazó rápidamente a todas las demás variantes circulantes de SARS- CoV-2 en el mundo. Esta variante llegó a representar cerca del 99 % de las infecciones por SARS-CoV-2 hasta mediados de noviembre del 2021 cuando apareció la variante ο que desplazó rápidamente a δ ^6^^,^^7^.

δ fue -entre agosto y diciembre de 2021- la variante responsable del mayor número de casos de COVID-19 a nivel mundial, debido a la presencia de mutaciones como L452R y P681R en la proteína *spike* que le confieren una mayor transmisibilidad respecto a otras variantes de SARS-CoV-2 ^8^^,^^9^. En marzo dle 2022, la Red Regional de Vigilancia Genómica de la Organización Panamericana de la Salud (OPS), en colaboración con los estados miembros, reportó 296.729 genomas δ para Latinoaérica y el Caribe en la plataforma GISAID (*Global Initiative on Sharing All Influenza Data*) ^6^.

En Colombia, el Instituto Nacional de Salud, a través del Programa Nacional de Caracterización Genómica del SARS-CoV-2, informó en noviembre del 2021 que la variante δ estaba representada en el 84,35 % de las secuencias del país ^10^. Antioquia es una de las poblaciones de Colombia con más secuencias reportadas en GISAID para enero de 2022 ^11^.

El *software* de asignación filogenética PANGOLIN (*Phylogenetic Assignment of Named Global Outbreak Lineages*) reporta 253 sublinajes de δ. De estos, la plataforma GISAID reporta 54 sublinajes en Antioquia con distribuciones diferenciales entre las subregiones ^12^.

La identificación y caracterización de los diferentes sublinajes δ permiten el seguimiento detallado del virus, la diferenciación de los sublinajes, el establecimiento de su origen y evolución, y el rastreo de cadenas de transmisión ^13^. Además, el seguimiento de mutaciones de interés en estos sublinajes -asociados con un mayor número de ingresos a unidades de cuidados intensivos, ventilación mecánica invasiva y muerte- es importante para fortalecer la implementación de las estrategias de control y mitigación que han demostrado disminuir los síntomas clínicos después de una infección por COVID-19 ^14^.

El presente estudio caracterizó la diversidad intragenómica de la variante δ mediante un análisis descriptivo y filogenético de las cepas virales circundantes a nivel local, regional y global. Se determinó la frecuencia de las mutaciones de interés que previamente se han asociado con infectividad, gravedad clínica y evasión del sistema inmunitario.

## Materiales y métodos

### Análisis descriptivo del contexto epidemiológico de las variantes de SARS-CoV-2

La información empleada para los análisis descriptivos y genómicos para la población de Antioquia hace parte de los muestreos probabilísticos definidos por el Instituto Nacional de Salud y por la vigilancia rutinaria de casos positivos que hacen parte de conglomerados, viajeros procedentes del exterior, hospitalizados, fallecidos y posibles reinfectados. Se recolectaron los datos sociodemográficos de 2.675 casos reportados en el Sistema de Vigilancia en Salud Pública (SIVIGILA) y se descargaron 1.115 genomas del repositorio GISAID ^15^.

Se empleó un análisis univariado para determinar la proporción de variantes de SARS-CoV-2 en Antioquia, su distribución temporal, proporción de casos fallecidos y hospitalizados, incidencia y tasa de letalidad.

### Secuenciación genómica de muestras de SARS-CoV-2

En Antioquia, el Laboratorio Departamental de Salud Pública, la Corporación de Investigaciones Biológicas y el laboratorio *One Health* de la Universidad Nacional de Colombia en Medellín conforman la Red Departamental de Vigilancia Genómica de SARS-CoV-2. Estas instituciones son las encargadas de secuenciar muestras clínicas (hisopado y aspirado nasofaríngeo) de los pacientes positivos.

Para la preparación de la librería y la secuenciación se siguió el protocolo *ARTIC network* empleando la tecnología de Oxford Nanopore ^16^^,^^17^. Se utilizó el kit de ligación SQK-LSK109 y para la marcación de las muestras se empleó el kit de códigos de barras EXP-NBD196. La librería fue cargada en una celda de flujo R9.4.1 en un secuenciador MinION Mk1C ^17^^,^^18^. Para este protocolo se empleó la versión 3 del esquema de cebadores (*primers*) para SARS-CoV-2.

Todas las secuencias con profundidad promedio mayor de 150 veces y más del 90 % de cobertura se cargaron en la plataforma GISAID. Los genomas de población antioqueña cargados a la plataforma GISAID por la Red de Vigilancia Genómica se descargaron para su posterior análisis con secuencias correspondientes a otras variantes reportadas en Colombia, como α, ɣ, μ y ο.

### Prevalencia de la variante δ y sus sublinajes en Antioquia

Para determinar el contexto epidemiológico en el que se identificó la variante δ y sus sublinajes en el departamento de Antioquia, se estimó su prevalencia en función del tiempo (12 de diciembre del 2020 al 31 de enero del 2022) y luego, la prevalencia de cada uno de los sublinajes para determinar cuál era el más representativo. Esto permitió determinar la circulación de los sublinajes δ a nivel regional y su contexto epidemiológico. Para este análisis se utilizó el *software* SPSS®, versión 26.0 ^19^.

### Selección de genomas y análisis filogenético

Se descargaron 1.115 genomas de δ reportados para Antioquia en la base de datos GISAID y se utilizaron para realizar los análisis descriptivos. Para el análisis filogenético se seleccionaron 501 genomas δ que cumplían con los criterios de calidad de cobertura superior al 90 % y profundidad mayor de 150 veces ^15^.

Las secuencias de los sublinajes δ se evaluaron en el contexto de la diversidad genómica global. Se realizó una comparación entre los genomas de los sublinajes δ con otras variantes de SARS-CoV-2, como α (50 genomas), γ (37 genomas), μ (40 genomas) y ο (45 genomas), descargados de la base de datos GISAID que también cumplieron con los criterios de calidad mencionados. Estos genomas se utilizaron para evaluar la adecuada asignación de sublinajes δ en el árbol filogenético y también para realizar análisis comparativos entre los sublinajes δ y otras variantes de SARS- CoV-2.

Se usó el *software* Muscle®, versión 5, para la alineación de todas las secuencias y se usó Wuhan-Hu-1 (GenBank: MN908947.3) como genoma de referencia ^20^.

Los extremos de las secuencias se eliminaron con el *software* MEGA 11® con el objetivo de que todos los genomas analizados tuvieran la misma longitud. Los *gaps* se eliminaron con Trimal®, versión 1.2 ^21^^,^^22^.

Las secuencias con valores atípicos en el árbol filogenético se eliminaron porque indicaban una baja calidad de alineación. También se eliminaron las secuencias duplicadas para evitar politomías.

La generación de árboles filogenéticos de máxima verosimilitud se estimó utilizando el *software* IQtree®, versión 2.1.1, bajo un modelo de sustitución de nucleótidos GTR + F + I según los criterios de información de Akaike (AICc) y Bayes (BIC) ^23^. Se utilizaron como medidas de soporte *Ultrafast Booptrap Aproximation* (UFB) con 10.000 réplicas y *approximate likelihood ratio test* (aLRT) con 1.000 réplicas. La visualización y edición del árbol se realizó con el programa FigTree®, versión 1.4.3 ^24^.

### Caracterización de mutaciones en los sublinajes δ

La estimación de las frecuencias alélicas para las mutaciones de interés se realizó utilizando Biophyton en un sistema operativo Linux y la generación de las gráficas se realizó en Microsoft^®^ Excel ^25^.

## Resultados

### Contexto epidemiológico de la variante δ

**Figura 1:**
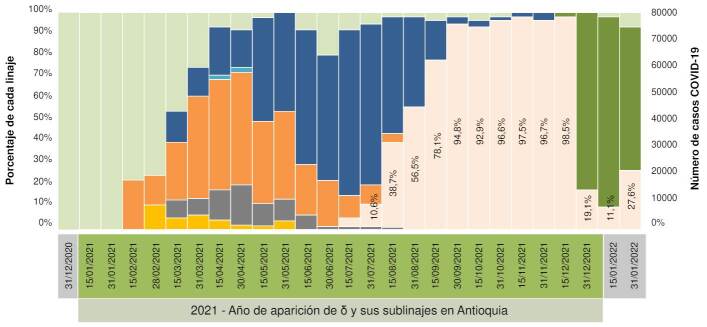
Porcentaje de linajes de SARS-CoV-2 en Antioquia, 2021-2022. Se observa como las variantes ɣ (naranja) y μ (azul oscuro) fueron las más prevalentes durante la primera mitad del 2021 (tercer pico de la pandemia) hasta la aparición de δ (naranja claro) en la segunda mitad del mismo año. δ desplazó a todas las demás variantes de SARS-CoV-2 y se convirtió en el linaje más representativo de este virus hasta diciembre del 2021 cuando apareció ο (verde oscuro), que desplazó rápidamente a δ durante ese mismo mes.

Los análisis descriptivos revelaron que, en Antioquia, la variante δ tuvo su primera aparición el 12 de julio del 2021. Para septiembre de ese mismo año se estableció como la variante predominante con 78,11 % de representatividad entre todas las muestras positivas para COVID-19 (figura 1). Para finales de noviembre de ese mismo año, δ estaba presente en más del 90 % de las muestras positivas hasta inicios de diciembre, cuando apareció ο y la desplazó rápidamente (figura 1). También se pudo evidenciar que, a pesar de la reciente aparición de ο, el 10 de diciembre del 2021, para el 31 de diciembre ya estaba presente en el 80,91 % de las muestras secuenciadas (figura 1).

**Figura 2:**
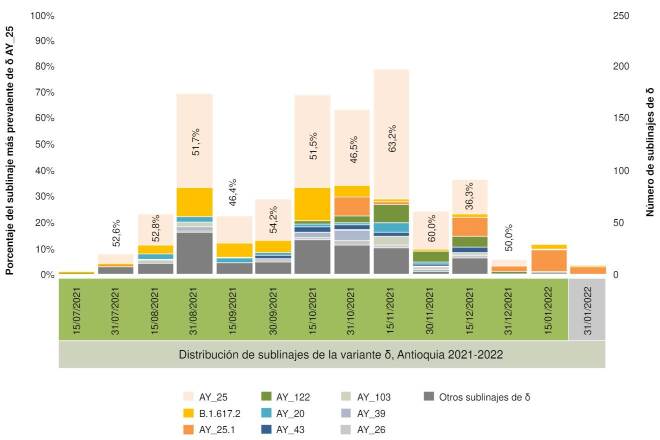
Conteo absoluto de sublinajes δ en Antioquia, 2021-2022. Se descargaron 1.115 genomas de la base de datos GISAID entre el 12 de julio del 2021 y el 18 de enero del 2022 que permitieron identificar 54 sublinajes δ: AY.25 (naranja pastel) fue el más prevalente desde julio hasta diciembre del 2021.

En el total de genomas analizados (1.115) se observó la existencia de una gran diversidad de sublinajes δ con 54 tipos identificados, el más prevalente de estos fue AY.25 (50,13 %), seguido de B.617.2 (10,49 %) y AY.25.1 (6,73 %) (figura 2). También se observó que entre el 30 de septiembre y el 30 de noviembre del 2021, el sublinaje AY.25 representó en promedio el 53,04 % de todas las secuencias de δ reportadas para ese periodo y que su presencia disminuyó significativamente a partir del 10 de diciembre del 2021 cuando aparece ο (figura 2). A partir del 15 de octubre de 2021, la Red de Vigilancia Genómica, con apoyo del Instituto Nacional de Salud, recibió una dotación de equipos MinION Mk1C para el fortalecimiento de la Red y con ello aumentar el número de muestras por secuenciar, lo que se vio reflejado en el incremento de genomas obtenidos a partir de esa fecha.

### Indicadores epidemiológicos relacionados con SARS-CoV-2 durante 2021

Los indicadores epidemiológicos mostraron que, durante el primer semestre del 2021, específicamente en los meses de marzo y julio, se presentó el tercer pico de la pandemia. En ese momento, los linajes μ y γ representaban el mayor porcentaje de variantes de SARS-CoV-2 entre las muestras positivas (cuadro 1). Indicadores como el porcentaje de positividad (23,77 %), la incidencia (5.662,71 por 100.000 habitantes), el número de hospitalizados (22.724), la tasa de mortalidad (139,27 por 100.000 habitantes) y la letalidad (2,46 %) fueron mayores en el segundo semestre del 2021 cuando δ fue la variante de SARS-CoV-2 predominante en la región (cuadro 1).

**Cuadro 1:** Indicadores epidemiológicos de SARS-CoV-2 por semestre y según linajes con mayor participación, Antioquia, 2021

Indicadores epidemiológicos	Primer semestre Circulación de μ/ɣ	Segundo semestre Circulación de sublinajes δ	Total	
Pruebas (n)	1’286.428	1’615.948	2’902.299	
Positividad (%)	11,78	23,77		18,45
Casos positivos (n)	151.527	384.078	535.605	
Incidencia por 100.000 habitantes	2.234,06	5.662,71		7.896,77
Hospitalizados (n)	6.870	22.724	29.556	
Proporción de casos hospitalizados en sala general	65,78	68,80		68,06
Proporción de casos hospitalizados en unidad de cuidados intensivos	34,22	31,20		31,94
Fallecidos (n)	2.217	9.446	11.663	
Tasa de mortalidad por cada 100.000 habitantes	32,69	139,27		171,96
Tasa de mortalidad	1,46	2,46		2,18

### Análisis filogenético

**Figura 3:**
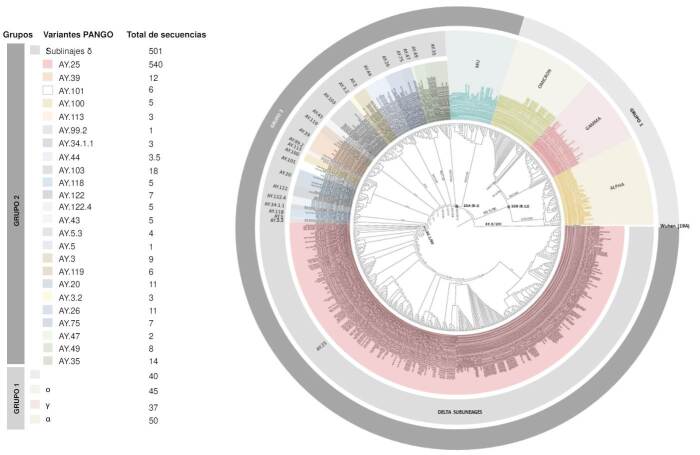
Árbol filogenético con sublinajes delta y otros ciados como ο, α, γ y μ. Se observa la amplia diversidad de sublinajes (n=54) presentes en el linaje δ a partir del análisis de 515 genomas seleccionados según criterios de calidad específicos. Este análisis reveló la gran representatividad del sublinaje AY.25 (50,13 %) (rosado). Se aprecian dos *clusters* según los ancestros comunes de los linajes SARS-CoV-2 que los anteceden. El primero está conformado por los linajes ο, α y γ, y el segundo grupo está definido por los linajes μ y los sublinajes δ.

En los 501 genomas que cumplieron los criterios de calidad para llevar a cabo el análisis filogenético se identificaron 24 sublinajes δ entre los que AY.25 fue el más predominante con el 67,86 %. El resto de los sublinajes no superó el 4 % en la muestra total analizada (figura 3).

La topología del árbol filogenético mostró dos grupos: el primero está definido por los linajes ο, α y ɣ; y el segundo, por los linajes μ y los sublinajes δ. Para el primer grupo se evidencia que las medidas de soporte aLRT/UFB de 90,5/95 indican con valores superiores al 90 % que las variantes α, ɣ y ο tienen un grado significativo de similitud genética que los diferencia, en cierto grado, del grupo dos, conformado por μ y los sublinajes de δ. Además, también se observa que, según la topología del árbol y los valores de aLRT/ UFB, el ancestro común de los tres linajes del grupo 1 es el clado 20B (B.1.1), del cual deriva primero la variante α y de ella, ɣ y ο. La separación bipartita que divide a ɣ de ο comprende un aLRT del 79,3 % y un UFB del 94 %. Dado que la métrica de soporte para la división de estas dos variantes comprende un UFB mayor del 90 %, se sustenta que dicha división es confiable. La topología del árbol también es confiable con relación a la separación de μ y los sublinajes δ (aLRT/UFB = 78,1/100), quienes tienen como ancestro común el clado 20A (B.1) (figura 3). Otra particularidad que muestra el árbol es la separación del sublinaje AY.25 (aLRT/UFB = 90,1/80), resaltado en color rojo, del resto de los sublinajes δ: se aprecia que AY.25 es el sublinaje más reciente o de más tardía aparición y además, es el más prevalente de los sublinajes δ (67,86 %) (figura 3).

### Frecuencias alélicas de mutaciones de interés en los linajes de SARS-CoV-2

En el análisis de frecuencias alélicas se observaron dos mutaciones en el gen que codifica para la proteína *spike*, L452R y P681R, presentes en el sublinaje AY.25 con una frecuencia de 0,985 y 0,997, respectivamente. L452R y P681R hacen parte de un grupo de mutaciones que definen el linaje δ y, por tanto, no se identificaron en las variantes α, ɣ, μ ni ο.

**Figura 4:**
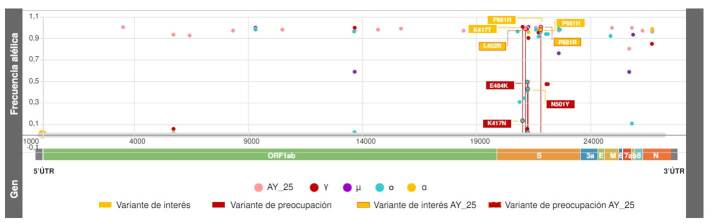
Estimación de las frecuencias alélicas de mutaciones de interés (rectángulo amarillo) y preocupación (rectángulo rojo) en el sublinaje AY.25 (rosado) versus ο (azul claro), γ (rojo), α (amarillo) y μ (morado). Las variantes de preocupación L452 y P681R restringen su presencia a AY.25 y comprenden una frecuencia de 0,99.

Otras mutaciones de interés epidemiológico asociadas con mayor transmisibilidad, como P681H, K417N, N501Y y E484K, no se encontraron en ninguno de los sublinajes δ, pero sí fueron identificadas en las otras variantes de SARS-CoV-2. P681H fue la única de las cuatro mutaciones que se encontró en α, ɣ, μ y ο, cuya frecuencia alélica fue cercana a 1,0 para todos los clados. Por otro lado, las tres mutaciones restantes se encontraron únicamente en ο en frecuencias de 0,43 para N501Y, 0,50 para E484K y 0,13 para K417N (figura 4).

## Discusión

En el primer semestre del 2021 se observó cocirculación de diferentes variantes del virus SARS-CoV-2, en mayor proporción μ y γ, que coincidió con el incremento del número de hospitalizados y fallecidos por COVID-19. Por esta época se observaron dos picos importantes: el primero, entre la semana 14 y 16 con más de 21.000 casos, y el segundo, entre las semanas 24 y 26 con más de 24.000 casos.

La variante δ fue reportada por primera vez en una muestra tomada el 12 de julio del 2021 y progresivamente alcanzó una representatividad de más del 90 % entre las muestras secuenciadas para el segundo periodo del 2021. Sin embargo, se observó un descenso importante de δ a mediados de diciembre del mismo año con la aparición de la variante ο, que rápidamente se diseminó en el departamento y con la que aumento el número de casos positivos de COVID-19. En las primeras tres semanas del 2022 se llegaron a reportar 4.289 casos según los datos del Laboratorio Departamental de Salud Pública de Antioquia ^4^.

Tanto el análisis filogenético como el epidemiológico revelaron gran representatividad y diversidad de sublinajes δ en Antioquia durante el segundo semestre del 2021. Estos datos tienen correspondencia con los reportados en 2023 por Jiménez-Silva y colaboradores, quienes evidenciaron el aumento exponencial de δ durante la segunda mitad del 2021 en algunas localidades de Colombia, como Bogotá, Cali y Córdoba, a partir del análisis de 610 muestras secuenciadas mediante el protocolo de Oxford Nanopore y ARTIC Network ^26^.

En los resultados de este estudio se observa que AY.25 fue el sublinaje de δ más prevalente, hallazgo similar a lo reportado por otros estudios previos como el de Murall *et al*. Los autores analizaron 1,5 millones de genomas δ clasificados por PANGOLIN y encontraron que el sublinaje AY.25 era el más prevalente en las provincias canadienses y presentaba un aumento exponencial en el segundo semestre de 2021, como ocurrió en Antioquia ^27^. Brown y colaboradores demostraron que para octubre del 2021, más del 80 % de los genomas eran AY.25, particularmente en los estados del sur, medio oeste y oeste de los Estados Unidos ^28^.

El análisis filogenético muestra una relación evolutiva entre el linaje μ y los sublinajes δ, concordante con la filogenia informada en las bases de datos de Nextrain. Estos clados tienen a 20A (B.1) como ancestro común y refuerzan su agrupamiento a partir de una consistencia de rama de 78,1/100 (aLRT/UFB), mientras que ο, ɣ y α forman otro grupo que tiene a 20B (B.1.1) como ancestro común ^29^ con una consistencia de rama de 79,3/94. La relación evolutiva entre δ y μ se sustenta en el grado de similitud genética que comparten entre sus genomas más que en su ancestro común 20A. Esta explicación se basa en varios estudios que han evidenciado la dinámica evolutiva del SARS-CoV-2 como perfiles mutacionales característicos de cada linaje que explican cierto grado de relación entre ellos. Este estudio expone que las mutaciones de μ y δ esclarecen parcialmente la similitud genética entre los linajes y, por ello, se agrupan en el árbol filogenético. No hay reportes que soporten la posibilidad de que δ derive de μ. Sin embargo, como se mencionó, los resultados de esta investigación son congruentes con los datos reportados por Nextrain, que denota el origen de δ y μ en el clado 20A.

Mediante la observación de las prevalencias del pool de muestras se pudo evidenciar que AY.25 tiene la mayor representatividad entre los sublinajes δ con el 67,86 %. Esto puede ser un indicador de que AY.25 tiene una ventaja de transmisión en comparación con los otros sublinajes δ, relacionada también con la presencia de mutaciones como L452R y P681R. Estas mutaciones se han asociado con mejor evasión de la respuesta inmunitaria y menor eficacia de los medicamentos. La frecuencia alélica reportada en este estudio para L452R fue del 0,985 y para P681R fue del 0,997. Las dos mutaciones fueron más prevalentes en AY.25 que en cualquiera de los otros sublinajes δ. L452R es una mutación contrasentido (*missense*) que implica el cambio de timina por guanina en la posición 22.917 del gen *spike*. Esta región de *spike* es responsable de codificar la subunidad S1 que forma parte del dominio de unión al receptor ACE2 de las células huésped. Los estudios funcionales han demostrado que esta mutación mejora la afinidad del dominio de unión de *spike* por el receptor ACE2 porque aumenta la expresión de la proteína y le da mayor estabilidad. También se ha demostrado que esta mutación aumenta la infectividad al promover la complementariedad electrostática entre el dominio de unión de la proteína *spike* con el receptor ACE2 y también contribuye a un aumento significativo en la replicación viral ^30^^-^^32^. Otras investigaciones, como la de Deng *et al*., en el 2021, demostraron a través de estudios *in vitro* -con líneas celulares humanas 293T y organoides pulmonares de las vías respiratorias humanas- que la mutación L452R aumenta significativamente la infectividad hasta 22,5 veces en la línea celular 293T y hasta 14,7 veces en los organoides en comparación con el nivel de infectividad de estas mismas líneas celulares portando la mutación D614G, también relacionada con una mayor infectividad ^33^.

En el caso de P681R, esta es una mutación sin sentido que comprende el cambio de una citosina por una guanina en la posición 23.604. Se encuentra en el gen spike, en la región que codifica para la subunidad S2 y que permite la fusión de la membrana viral con la membrana de la célula huésped. Específicamente, esta mutación ocurre en el sitio de escisión de furina donde se da la separación de la subunidad S1 y S2 de la spike. Posterior a esto, se lleva a cabo la fusión de la membrana de la célula huésped con la membrana viral. Se ha demostrado que la presencia de P681R aumenta significativamente la replicación del virus ^34^^,^^35^.

En el resto de los linajes no se detectó P681R y en su lugar se encuentra en la misma posición (681) una variante que incluye un cambio de aminoácido definido como P681H (prolina por histidina). Esta modificación, al igual que P681R, se encuentra en el sitio de escisión proteolítica de la furina, por lo que también se ha relacionado con la modulación de la división del complejo S1/ S2 y, por lo tanto, aumenta las propiedades infecciosas del virus ^36^.

Entre otras mutaciones, es importante destacar la N501Y asociada con una mayor resistencia a fármacos, como el bamlanivimab, que proporcionan inmunidad humoral pasiva para reducir la carga viral durante la etapa inicial de la infección ^37^.

En el caso de la variante ο, se encontró la mutación E484K que restringe su presencia a este clado, sin encontrarla en ninguna de las otras variantes de SARS-CoV-2. Esta mutación está relacionada con la interacción de fármacos cuyo principio activo son anticuerpos monoclonales que inhiben la unión entre el dominio de *spike* y ACE2. El aminoácido E484 (ácido glutámico), presente en el genotipo “silvestre” de *spike* forma tres puentes salinos con las argininas de fármacos como el bamlanivimab dada la carga negativa de este aminoácido y la carga positiva de las argininas. Esto permite la acción del anticuerpo monoclonal que consiste en inhibir la unión del dominio de spike con el receptor ACE2 de la célula huésped. La mutación E484K impide la unión a bamlanivimab ya que la lisina de la posición 484 está cargada positivamente y repele su unión a los residuos de arginina del fármaco. Esto ha permitido que linajes como ο tengan la capacidad de evadir la respuesta inmune y, junto con otras mutaciones presentes como K417N y N501Y, ser más transmisibles ^38^. Algunos estudios demuestran que la mutación E484K reduce la unión de anticuerpos policlonales hasta 10 veces ^39^.

Como conclusión, es posible decir que la rápida y prolongada circulación de linajes del virus SARS-CoV-2 ha favorecido la aparición de nuevas mutaciones en su material genético. Un ejemplo de esto es la amplia diversidad de sublinajes δ presentes en Antioquia. La vigilancia genómica de estas mutaciones y los diferentes linajes que han venido evolucionando es de gran importancia ya que permite identificar y caracterizar brotes, controlar epidemias y tomar las medidas pertinentes de salud pública.
